# Silicon mitigates nutritional stress of nitrogen, phosphorus, and calcium deficiency in two forages plants

**DOI:** 10.1038/s41598-022-10615-z

**Published:** 2022-04-22

**Authors:** William Bruno Silva Araújo, Gelza Carliane Marques Teixeira, Renato de Mello Prado, Antonio Márcio Souza Rocha

**Affiliations:** 1 Federal Institute of Education, Science and Technology of Pará (IFPA)-Rural Campus of Maraba, Marabá, Pará Brazil; 2 Department of Agricultural Sciences, São Paulo State University (Unesp), Faculty of Agricultural and Veterinary Sciences (FCAV), Jaboticabal, São Paulo Brazil; 3 Department of Technology, São Paulo State University (Unesp), Faculty of Agricultural and Veterinary Sciences (FCAV), Jaboticabal, São Paulo Brazil

**Keywords:** Plant development, Plant physiology, Plant stress responses

## Abstract

Forages are one of the most cultivated crops in the world. However, nutritional deficiency is common, specifically in N, P, and Ca in many forage-growing regions. Silicon (Si) can attenuate the stress caused by nutritional deficiency, but studies on Si supply’s effects on forage plants are still scarce. This research was carried out to evaluate whether the Si supply can mitigate the effects of N, P, and Ca deficiencies of two forages and the physiological and nutritional mechanisms involved. Two experiments were carried out with two forage species (*Urochloa brizantha* cv. Marandu and *Megathyrsus maximum* cv. Massai). We used nutrient solution under balanced nutrition conditions and nutritional stress due to the lack of N, P, and Ca combined with the −Si and +Si. The deficiencies of N, P, and Ca in both forages’ cultivation caused damage to physiological and nutritional variables, decreasing the plant dry matter. However, in both forage species, the Si addition to the nutrient solution decreased the extravasation of cellular electrolytes and increased the content of phenolic compounds, the green colour index, the quantum efficiency of photosystem II, the efficiencies of use of N, P and Ca and the production of shoot dry matter. The beneficial effects of Si were evidenced in stressed and non-stressed plants. The research emphasised the advantage of using Si to grow *U. brizantha* and *M. maximum* under N, P, and Ca deficiency, contributing to their sustainable cultivation.

## Introduction

Pastures occupy a vast cultivated area worldwide. This is because almost all beef production comes from animals fed only pasture due to its low cost of production^1^. However, pasture cultivation often takes place in low fertility soils and under inadequate nutritional management^2^. This fact is worrisome, as nutritional deficiency has a global impact on crop production and increases the seasonality of forage production^3^. The nutrient uptake of plants depends on soil nutrient availability, and lack of nutrients can affect essential components of plant metabolism^4^.

A strategy to mitigate the effects of nutritional deficiency in forages is the use of silicon (Si)^5^. Soil Si availability decreases in pasture areas due to degradation at different levels^6^, being aggravated by Si export via grazing without replacement. The main mechanisms of action of Si correlate with oxidative damage reduction, and effect on the biosynthesis of phenolic compounds^7^, thus increasing photosynthetic rate and nutrient uptake^8^.

The literature also indicates that Si can bind calcium-like compounds to organic compounds in the cell wall; thus, Si can occupy calcium-binding sites, further minimising the effects of Ca deficiency^9^. Other studies added that Si can replace part of the carbon in organic structures of the cell wall^8^ with low energy cost^10^, enhancing nutrient uptake and nutrient use efficiency^11^.

However, most of these beneficial effects have been recorded in plants under biotic stress^12^. Studies addressing abiotic stresses refer to damage by salinity^13^ and water conditions^11,14,15^. Regarding nutritional disorders, studies have focused on the benefit of Si in relieving nitrogen (N) deficiency in rice^16^, potassium (K) deficiency in sorghum^17^ and maize^18^, magnesium (Mg) deficiency in maize^19^, calcium (Ca) deficiency in cabbage^20^ and rocket^21^, phosphorus (P) deficiency in sorghum^22^, wheat^23^, and cucumber^24,25^.

The beneficial effects of Si are more evident in plants with high accumulation of this element, such as those of the Poaceae family, including forages^26^. Notwithstanding, studies evaluating the effect of Si on the mitigation of abiotic stresses in forage plants are still limited. One study indicates a positive effect of this element on the alleviation of water deficit damage in *Brachiaria* ‘Mavuno’^27^. Another study addresses nutritional deficiency in *Panicum maximum* and in the hybrid Ipyporã (*B. ruziziensis* × *B. brizantha*)^28^. Considering only the genera *Urochloa* and *Megathyrsus*, as far as we know there is no previous report on the use of Si to alleviate nutritional deficiencies^29^.

These forages present low yield in tropical regions, especially due to deficiency of macronutrients, such as N, P, and Ca^30^. It is not yet known whether Si could contribute to mitigate nutritional deficiency in these species, or the mechanisms of action by which it would occur. However, this information would be important to increase the sustainability of tropical forage production systems, as these species are mostly grown in low fertility soils.

In this context, the hypothesis arises that Si can alleviate the effects of N, P, and Ca deficiency in forage crops. Thus, this research assesses whether Si application can alleviate the effects of N, P, and Ca deficiency in two forages, as well as the physiological and nutritional mechanisms involved.

## Material and methods

### Experimental conditions

Two experiments were conducted simultaneously in a greenhouse located at UNESP, Campus de Jaboticabal, from January to June 2019. First, data on relative air humidity and maximum and minimum temperature inside the greenhouse were recorded daily with the aid of a thermohygrometer (U23-001, Sigma Sensors, Brazil). As a result, there was a variation in the relative air humidity (60 ± 15%), maximum temperature (40 ± 10 °C), and minimum temperature (32 ± 10 °C).

We used two forage species *U. brizantha* cv. Marandu (Experiment 1) and *M. maximum* cv. Massai (Experiment 2), obtained from the Brazilian Agricultural Research Corporation of the Ministry of Agriculture, Livestock, and Food Supply. All plant studies were carried out following relevant institutional, national, or international guidelines and regulations. Our research was not conducted with endangered species and was conducted following the Declaration of IUCN Policy on Research Involving Endangered Species.

Sowing was carried out in polypropylene trays containing vermiculite as a substrate. The plants were kept in the trays for 20 days, receiving only distilled water. After this period, the seedlings were selected, washed, and transplanted to plastic pots with a volume of 1.7 dm^3^, filled with previously washed sand of medium size. After transplanting the plants, the application of the complete nutrient solution was carried out as indicated by Hoagland and Arnon^31^, with ionic strength of 25%, over 4 days. Then, the nutrient solution was applied following the treatments with ionic strength adjusted to 50% until the end of the experiment. The ionic strength variation was performed to avoid salinity stress to the plants.

The nutrient solutions were prepared with distilled and deionised water. We changed the iron source from Fe-EDTA to Fe-EDDHMA using double the dose as recommended^32^. The pH value was maintained at 5.5 ± 0.2 and monitored daily with a digital pH meter (ICEL PH-1500). When necessary, it was adjusted with HCl or NaOH solution.

### Experimental design and treatments

The experiment was carried out in a 4 × 2 factorial scheme. The treatments consisted of control (complete solution—CS); solution deficient of N (−N), P (−P), and Ca (−Ca); combined without (−Si) and with of Si (+Si). Treatments were arranged in experimental design was completely randomised, with six replicates.

The nutrient solution for the treatments was prepared according to Hoagland and Arnon^31^. For treatments with −N (deficient N), a deficiency equivalent to 50% of the maximum dry matter production was simulated, associated with 6 mmol L^−1^ of N^33^. Nitrogen was supplied in ammonium nitrate (NH_4_NO_3_) at a concentration of 3.0 mmol L^−1^ for 3 days, followed by 4.5 mmol L^−1^ for the same period and 6.0 mmol L^−1^ until the end of the experiment (Table Supplementary 1).

In treatments with −P, this nutrient was initially omitted from the nutrient solution and, after 30 days, 0.05 mmol L^−1^ was added until the experiment’s end. For treatments with −Ca, the nutrient was omitted from the beginning until the experiment’s end (Table Supplementary 1).

The supply of Si was started after 5 days of emergence, applying only to the +Si treatment. The Si source was sodium silicate stabilised with sorbitol (94.2 g L^−1^ of Si and 60 g L^−1^ of Na, pH 12.6), at a concentration of 2.0 mmol L^−1^, supplied via nutrient solution (via root). Silicon was added to the nutrient solution, and the pH value was adjusted to 5.5 ± 0.2 with Hydrochloric acid (HCl) or Sodium hydroxide (NaOH) solutions, and it was immediately supplied to the plants. The additional 49.8 mg L^−1^ of NaCl application was performed in the treatments −Si to balance the Na among treatments.

Once a week, the substrate was washed to avoid salinisation, and deionised water was applied to the substrate until drainage. The nutrient solution was applied again after 2 h. Forty-five days after transplanting, a standardisation cut was performed in the forages at the height of 5 cm from the substrate.

### Analyses

Ninety days after transplanting, with the expression of classic signs of nutrient deficiency, the electrolyte leakage index, green colour index, and quantum yield of photosystem II were evaluated. These evaluations were carried out on the second fully expanded leaf in the treatments with −N and −P and the first fully expanded leaf for −Ca. In the CS plants, we also collected data for these two leaves.

#### Green colour index

According to the treatment, the green colour index was measured with the aid of Opti-sciences^®^ equipment—CCM-200, from three readings carried out on the fully expanded leaves. We used the second fully expanded leaf in the treatments with −N and −P, on the first fully expanded leaf for −Ca, and on both leaves in the CS.

#### Quantum yield of photosystem II (Fv/Fm)

Quantum yield of photosystem II (Fv/Fm) was measured in the morning (7 to 8 a.m.), using a fluorometer device (Opti-sciences^®^—Os30P+). For this measurement, we placed the sampled region in the dark for adaptation at least 30 min before the excitation of the red-light pulse of 1 s^34^. Readings were taken in the middle third of the leaf, avoiding the midrib. We used the second fully expanded leaf in the treatments with −N and −P, on the first fully expanded leaf for −Ca, and on both leaves in the CS.

#### Electrolyte leakage index

For determining the electrolyte leakage index, ten leaf discs (6 mm) were removed from the middle third of the second fully expanded leaf in the treatments with −N and −P, on the first completely expanded leaf for −Ca, and on both leaves in the CS.

The discs were packed in a beaker with 20 mL of deionised water at ambient temperature for 2 h. After this period, the initial electrical conductivity reading (EC_1_) was performed using a manual conductivity meter (TDS-3). Afterwards, samples were taken to the autoclave for 20 min at a temperature of 121 °C. After cooling, a new conductivity reading was performed to determine the final conductivity (EC_2_). In order to estimate the electrolyte leakage index, the following formula was used: EC_1_/EC_2_ × 100^35^.

#### Phenolic compounds

Quantification of total phenols content was performed in the middle portion of the second fully expanded leaf using the method described by Singleton et al.^36^. For such, we emerged samples of fresh leaves in concentrated methanol in a water bath at 25 °C. After extraction, a colourimetric reaction of total phenols was induced with the 2 N Folin–Ciocalteu reagents, allowing reacting for 3 min, and 20% sodium carbonate, allowing reacting for 2 h. In the end, the absorbance was read on a spectrophotometer at a wavelength of 765 nm, while the content was determined using a standard curve with gallic acid, expressed as Gallic acid equivalent (GAE) 100 g^−1^.

#### Plant length and number of tillers

At 55 days after the forage uniformity cut, we measured the length of the plants from the base to the top of the plant using a measuring tape. On this day, all forage tillers were counted.

#### Dry matter

At 55 days after the uniform cut, the plants were harvested then washed in running water with a detergent solution (0.1% v/v), HCl solution (0.3% v/v), and twice in deionised water. Subsequently, the plant material was oven-dried with forced air circulation (65 ± 5 °C) until it reached constant mass. Then, the shoot dry matter of the forages was obtained.

#### Si accumulation

The Si content was determined in samples obtained from shoots and roots of forages. For such, 0.1 g of dry and ground material was added to 50 mL polyethene tubes. The samples were moistened with 2 mL of hydrogen peroxide (H_2_O_2_), the tube was placed in an oven at 95 °C. After 30 min, the tubes were removed, and 4 mL of 50% NaOH were added to warm samples. The sample tubes were then gently vortexed and returned to the oven (95 °C) for 4 h according to the methodology described in Kraska et al.^37^. The Si concentration was determined by colourimetry using 1 mL extract plus 19 mL of water, 1 mL of HCl (1:1), and 2 mL ammonium molybdate. After 5 min, 2 mL of oxalic acid were added. The reading was performed by a spectrophotometer at 410 nm as described in Korndörfer et al.^38^.

#### N, P and Ca accumulation and use efficiencies

The N content was determined by adding concentrated sulfuric acid to previously dried and ground plant material samples, followed by distillation and titration with sulfuric acid^39^. The levels of P and Ca were determined by the digestion of samples of plant material, using a digestive mixture of perchloric and nitric acid (1:2), with readings of Ca performed in spectrophotometry atomic absorption with air-acetylene flame and P readings through spectrophotometry^39^.

Based on the content and shoot dry matter, the accumulation of each nutrient in the shoot of the forages was obtained to be used in the calculation of the N, P, and Ca use efficiencies, according to the equation: (shoot dry matter)^2^/nutrient accumulation in shoot^40^.

### Statistical analysis

The data obtained were submitted to analysis of variance by the *F* test. If significant (*p* < 0.01) and for differentiation between treatments, Tukey’s means comparison test (*p* < 0.05) was performed using the SAS^®^ statistical software (Cary, NC, USA).

## Results

**Figure 1:**
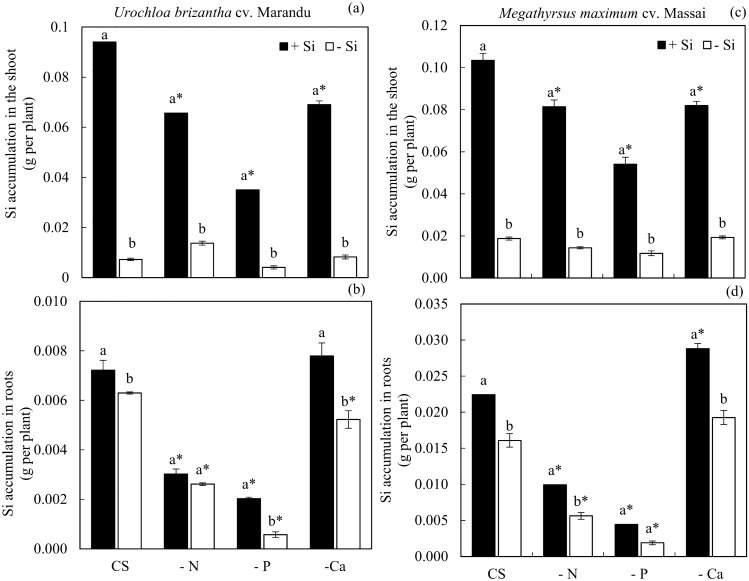
Silicon (Si) accumulation in the shoot (**a**,**c**) and roots (**b**,**d**) of *Urochloa brizantha* cv. Marandu and *Megathyrsus maximum* cv. Massai under grown in complete solution (CS) and deficiency of nitrogen (−N), phosphorus (−P) and calcium (−Ca), combined with Si (+Si) and without Si (−Si). Different letters indicate a significant difference between treatments in the +Si and −Si condition. *Indicates a significant difference between treatments with nutritional deficiencies in relation to treatments with CS, by the Tukey test (*p* < 0.05).

The accumulation of Si in the shoot decreased in plants grown in Si and under deficiencies in N, P, and Ca compared to the CS in *U. brizantha* cv. Marandu and *M. maximum* cv Massai (Fig. 1a,c). However, the addition of Si in both forage plants without and with a nutritional deficiency concerning −Si increased the accumulation of Si in the plant’s shoot (Fig. 1a,c).

The accumulation of Si in the plant roots decreased with deficiencies in N and P compared with CS, independent of Si in both species (Fig. 1b,d). In plants with Ca deficiency, this decrease was observed only in plants under the absence of Si in *U. brizantha* cv. Marandu, compared with the CS and −Si (Fig. 1b).

Silicon addition in plants of both species under CS, and with deficiencies in P and Ca, compared to the treatment without Si, increased the Si accumulation in the root (Fig. 1b). Si accumulation in the plants’ roots with deficiencies in N increased only in plants of *M. maximum* cv Massai (Fig. 1d) but did not differ in *U. brizantha* cv. Marandu regarding −Si (Fig. 1b).

**Figure 2:**
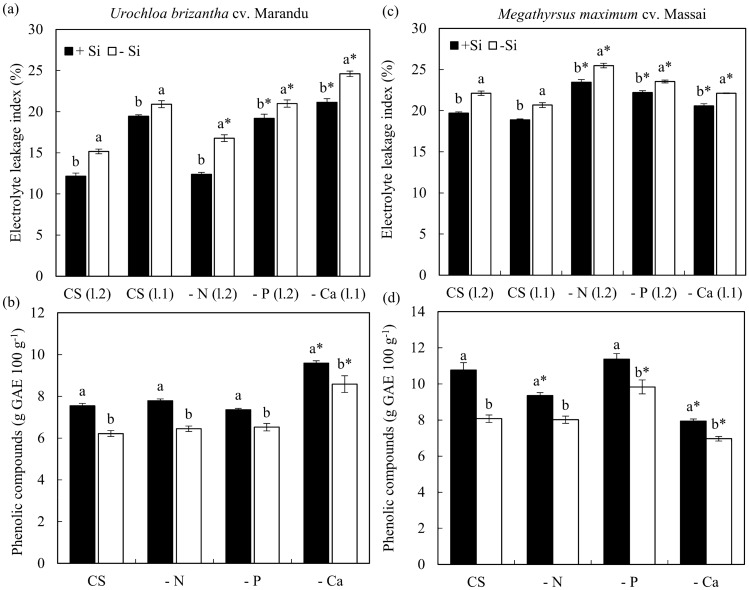
Electrolyte leakage index (**a**,**c**) and phenolic compounds (**b**,**d**) of *Urochloa brizantha* cv. Marandu and *Megathyrsus maximum* cv. Massai under grown in complete solution (CS) and deficiency of nitrogen (−N), phosphorus (−P) and calcium (−Ca), combined with Si (+Si) and without Si (−Si). Different letters indicate a significant difference between treatments in the +Si and −Si condition. *Indicates a significant difference between treatments with nutritional deficiencies in relation to treatments with CS. For the electrolyte leakage index the treatments of CS (l.2) are compared with the treatments −N (l.2), −P (l.2), refer to values obtained by the evaluation of the second fully expanded sheet. The treatments of CS (l.1) are compared with the values of −Ca (l.1) and refer to the first leaf. The comparison of means was performed using the Tukey test (*p* < 0.05).

Plants that received a solution deficient in P and Ca, independent of Si, compared to plants under CS, showed an increase in the electrolyte leakage index in both species (Fig. 2a,c). On the other hand, the supply of Si in all treatments (−N, −P, −Ca, and CS) compared to treatments that did not receive Si decreased the electrolyte leakage index (Fig. 2a,c). This effect was evident in *U. brizantha* cv. Marandu under N deficiency, in which the application of Si reduced electrolyte leakage index, making it similar to that obtained in plants under CS (Fig. 2a).

In *U. brizantha* cv. Marandu, the levels of phenolic compounds in plants grown in CS and solutions deficient of N and P were similar in each Si supply condition. Nevertheless, the content of these compounds increased in the solution deficient in Ca, independent of Si addition (Fig. 2b).

In *M. maximum* cv Massai, the levels of phenolic compounds decreased with Ca deficiency, independent of Si, and with N deficiency only in the presence of Si, compared to CS and +Si. Moreover, under P deficiency, the phenols content in *M. maximum* cv Massai increased regarding CS, but only in the −Si (Fig. 2d). The addition of Si increased the plant phenols content, regardless of whether it was grown in a CS solution or under −N, −P, and −Ca in both species (Fig. 2b,d).

**Figure 3:**
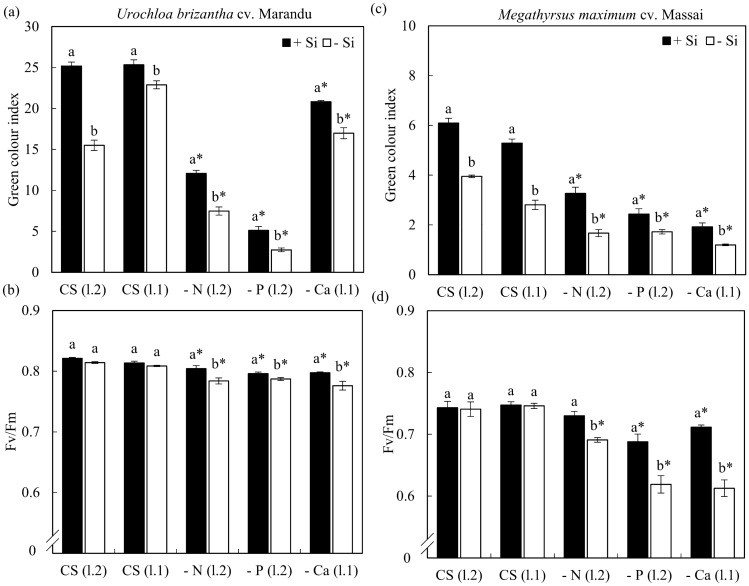
Green colour index (**a**,**d**) and Fv/Fm (**b**,**d**) of *Urochloa brizantha* cv. Marandu and *Megathyrsus maximum* cv. Massai under grown in complete solution (CS) and deficiency of nitrogen (−N), phosphorus (−P) and calcium (−Ca), combined with Si (+Si) and without Si (−Si). Different letters indicate a significant difference between treatments in the +Si and −Si condition. *Indicates a significant difference between treatments with nutritional deficiencies in relation to treatments with CS. The treatments of CS (l.2) are compared with the treatments −N (l.2), −P (l.2), refer to values obtained by the evaluation of the second fully expanded sheet. The treatments of CS (l.1) are compared with the values of −Ca (l.1) and refer to the first leaf. The comparison of means was performed using the Tukey test (*p* < 0.05).

The green colour index decreased in forage cultivated with a solution deficient in N, P, and Ca, compared to the CS, independent of Si, in both species (Fig. 3a,c). However, in the two species, this index increased due to the increase of Si in plants deficient in N, P and Ca (Fig. 3a,c).

The fluorescence quantum yield (Fv/Fm) was lower when plants were grown under −P and −Ca, regarding CS, independent of Si in both species. Under −N, this decrease occurred in both Si conditions in *U. brizantha* cv. Marandu, but only in the absence of Si in *M. maximum* cv. Massai (Fig. 3b,d). The addition of Si increased the quantum yield of forage grown under −N, −P and −Ca regarding −Si but did not change this parameter for plants grown in the CS (Fig. 3b,d).

**Figure 4:**
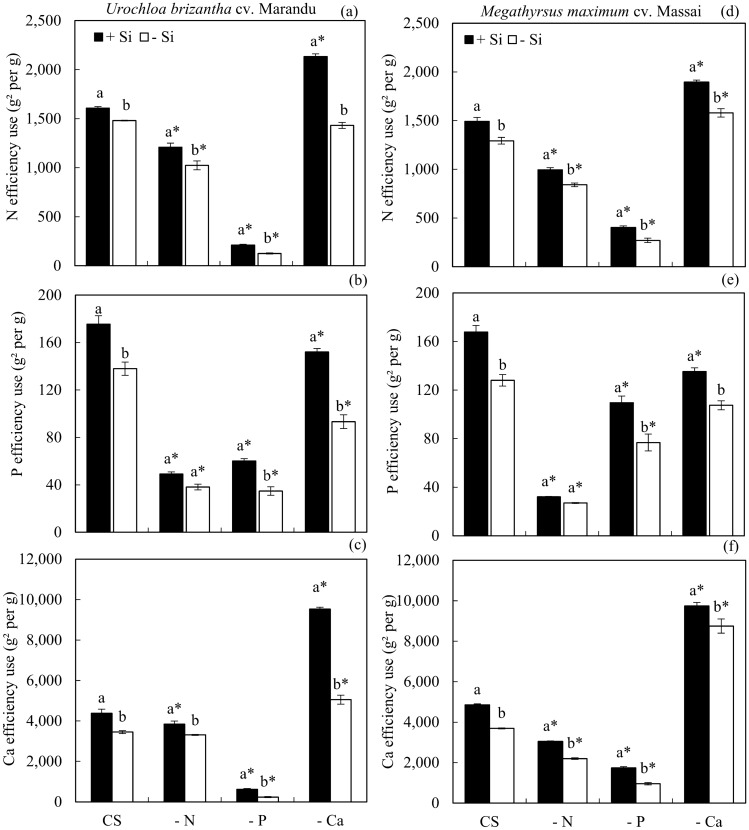
Efficiency of use the nitrogen (N) (**a**,**d**), phosphorus (P) (**b**,**e**) and calcium (Ca) (**c**,**f**) of *Urochloa brizantha* cv. Marandu and *Megathyrsus maximum* cv. Massai under grown in complete solution (CS) and deficiency of nitrogen (−N), phosphorus (−P) and calcium (−Ca), combined with Si (+Si) and without Si (−Si). Different letters indicate a significant difference between treatments in the +Si and −Si condition. *Indicates a significant difference between treatments with nutritional deficiencies in relation to treatments with CS, by the Tukey test (*p* < 0.05).

The N use efficiency was lower under nutrient deficiency, regarding CS, independent of Si in both species. However, it increased when plants were deficient in N and Si was added (Fig. 4a,d). The P use efficiency was lower under P deficiency, both in the +Si or −Si in the nutrient solution, regarding CS, in both species. However, it increased when Si was added (Fig. 4b,e). In both species, the Ca use efficiency was higher when plants were grown without Ca, independent of Si, compared to CS. The addition of Si increased this parameter (Fig. 4c,f).

**Figure 5:**
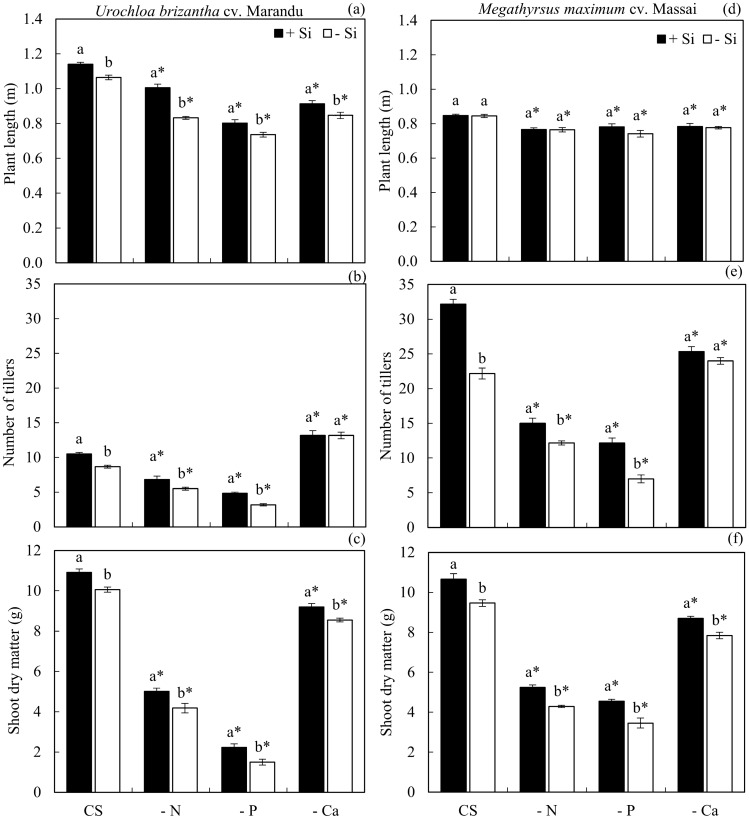
Plant length (**a**,**d**), number of tillers (**b**,**e**) and shoot dry matter (**c**,**f**) of plants of *Urochloa brizantha* cv. Marandu and *Megathyrsus maximum* cv. Massai under grown in complete solution (CS) and deficiency of nitrogen (−N), phosphorus (−P) and calcium (−Ca), combined with Si (+Si) and without Si (−Si). Different letters indicate a significant difference between treatments in the +Si and −Si condition. *Indicates a significant difference between treatments with nutritional deficiencies in relation to treatments with CS, by the Tukey test (*p* < 0.05).

Plant length decreased when plants were cultivated in solution deficient in N, P, and Ca regarding the CS, independent of Si, in both species. However, the addition of Si resulted in increased plant length, irrespective of the nutrient solution, but only in *U. brizantha* cv. Marandu (Fig. 5a,d).

Tiller number of plants cultivated in solution deficient of N and P decreased regarding the CS, independent of Si, in both species (Fig. 5b,e). However, in *U. brizantha* cv. Marandu under Ca deficiency in +Si and −Si, the number of tillers was higher than that of plants grown in CS, but for *M. maximum* cv. Massai, this occurred only in −Ca and −Si. The addition of Si to the CS and the solutions deficient in N and P increased the number of tillers in both species (Fig. 5b,e).

The shoot dry matter production was impaired in both forages cultivated in solution deficient in N, P, and Ca, irrespective of Si addition, compared to CS. In contrast, the addition of Si to the CS and the group deficient in N, P, and Ca increased the shoot dry matter production in both species (Fig. 5c,f).

## Discussion

### Silicon improves the performance of forage plants without nutritional deficiency

Silicon addition in the complete nutrient solution favoured shoot dry matter production in both forages under all conditions. There is little evidence of the beneficial effects of Si on plants grown without specific nutritional disorders. Nevertheless, some reports indicate the positive effects of Si on crops under biotic^12^ or abiotic stresses^14,17^. In the present study, the beneficial effect of Si on dry matter production was evident even in plants grown without nutritional deficiency. This Si effect occurred due to its positive effects on antioxidant capacity, evidenced by increased production of phenolic compounds. This in turn reduced the electrolyte leakage index, increasing green colour index, fluorescence quantum yield, and ultimately N, P, and Ca use efficiencies.

This effect of Si might also be because this element could decrease the demand for other compounds of the cell wall, such as lignin, which demands more energy^10^. Silicon can replace part of the carbon in the formation of some low energy cost structural compounds^8^, especially in the cell wall^26^. In grasses, the element concentrates mostly in leaf cell walls^10^, which may improve nutrient use efficiency, thus favoring biomass production. Previous reports show this effect for sugarcane^41^ and *Brachiaria* ‘Mavuno’^27^. However, confirmation of this hypothesis depends on specific studies on the cell walls of *U. brizantha* cv. Marandu and *M. maximum* cv Massai.

The beneficial effect even on plants without nutritional stress is due to the high accumulation of Si, above all, in the shoots of both forage species under study, showing their great capacity to absorb and accumulate this element^5^. This result indicates that Si can be used for the cultivation of forage species without nutritional deficiency to increase the sustainability of the cultivation of these species through a better management of macronutrient supply. Other studies show similar results for sugarcane^25^ and forage plants (*P. maximum* and the hybrid Ipyporã (*B. ruziziensis* × *B. brizantha*)^28^.

### Silicon mitigates nutritional N deficiency in forage plants

Plants grown in N-deficient nutrient solution showed impaired growth. This is due to the participation of N in the constitution of enzymes and root cell membrane transporters^42^. Nitrogen is involved in the active absorption of nutrients; thus, its deficiency affects this process negatively. Moreover, the low leaf N content directly interfered with pigments, as N is a structural nutrient of the chlorophyll molecule^30,43^.

This was evidenced by the decrease in leaf green colour index. Loss of pigmentation in leaves under N deficiency also has to do with the redistribution of this element due to proteolysis of the enzyme Rubisco and other chloroplast proteins, releasing the N present in these compounds to meet the demand for new organs^44^.

This decrease in nitrogen reduced the photosynthetic rate, as photosynthetic pigments play a key role in transferring excitation energy to photosystems^45^. The result was oxidative stress, evidenced by increased cellular electrolyte leakage. The reduction of phenolic compounds (which can act as nonenzymatic antioxidants) further aggravated stress^46^. These effects impaired plant growth and dry matter production.

On the other hand, Si supply physiologically improved plants under nutritional deficiencies. This was evidenced by the increase in phenolic compounds, green colour index, and fluorescence quantum yield (Fv/Fm). Other authors have reported similar results for barley^7^ and ryegrass^17,47^. In these studies, Si increased phenol production, thus improving the antioxidant system and increasing chlorophyll content as well as photosystem II efficiency.

Nitrogen-deficient plants that received Si showed better physiological conditions, with increased N and Ca use efficiencies and beneficial effects on plant growth and shoot dry matter production. Nutritional deficiencies can limit plant growth by causing imbalances and reducing nutrient use efficiency, which in turn decreases biomass production^42^. Thus, by improving nutritional efficiency, Si reduces the damage caused by N deficiency in these forage crops.

In this sense, the benefits of Si in mitigating the effects of N deficiency in the forages *U. brizantha* cv. Marandu and *M. maximum* cv Massai are unprecedented, like the results of a study investigating other grasses such as rice^16^.

Silicon application improved the performance of the two forage species under N deficiency. This suggests that the use of Si is a good strategy to enhance N fertilisation. This strategy has high sustainability since it preserves N, whose production depends on natural gas, a non-renewable source^48^. These results are important because the occurrence of low levels of N is common in forage cultivation areas^30^ due to the degradation scenario that reduces organic matter, an important source of N^1,48^.

### Silicon mitigates nutritional P deficiency in forage plants

Plants grown in P-deficient nutrient solution showed impairments in all variables under study. This is because P plays an important role in the energy metabolism of plants and in the formation of ATP and ADP^49^. Low P levels in the plant affect plant growth and development due to lower formation of new cells and lower cell elongation^50^. Moreover, P correlates with metabolic activities, and its low supply directly affects gas exchange, photosynthetic rate, and the activity of ribulose 1,5-bisphosphate carboxylase (Rubisco)^51^.

These effects increase the production of reactive oxygen species that degrade the cell membrane due to lipid oxidation^52^. This can be seen from the increased rate of electrolyte leakage and the decreased synthesis of phenolic compounds, as indicated by Ma et al.^53^ in wheat plants. The present study demonstrated this situation for the first time in the forage plants *U. brizantha* cv. Marandu and *M. maximum* cv Massai. However, further studies have demonstrated it in other grasses such as sorghum^22^, cucumber^24^, snap bean^54^, and quinoa^55^.

Silicon addition alleviated the damage of P deficiency, a fact not yet described in the literature for these species. This finding can be explained by Si-mediated improvements in the antioxidant system of these plants through the influence of this element on the metabolism of phenolic compounds. The plasma membrane was thus preserved, with lower electrolyte leakage index, which increased the green colour index. This suggests a higher chlorophyll content^56^, favouring photosynthetic efficiency, which results in energy (ATP) that is important for P transporters^57^.

Silicon can also increase the gene expression of P transporters, and this can favour P uptake by plants deficient in this nutrient^23^. Some reports have already described this effect for K, in which Si improved its absorption by the plant from the activation of H-ATPase^58^. The Si-mediated activation of nutrient transporting enzymes may explain the increase in P use efficiency in both forage species under study. The literature shows similar results for wheat^23^ and sorghum^22^ under P.

### Silicon mitigates nutritional Ca deficiency in forage plants

Cultivation under Ca deficiency reduced plant growth, green colour index, and fluorescence quantum yield. This fact was due to a lower expansion and lack of rigidity of the cell wall, which is governed by Ca^2+^ bonds to pectates^59^. The Ca within the cytosol can also act as a signal for several enzymes related to photosynthesis and as a secondary messenger in both biotic and abiotic stresses^59^, contributing to the decrease in dry matter accumulation. In addition, for both forages under study, Ca-deficient plants that did not receive Si in the nutrient solution presented a decrease in Ca use efficiency, which decreased dry matter production.

Notwithstanding, these negative impacts were mitigated with the addition of Si. This beneficial effect of silicon on plants was evidenced by its accumulation in the shoots of Ca-deficient plants, which increased the production of phenolic compounds. Other authors have also reported this effect for ryegrass^47^, barley^7^, and rocket plants^21^. The increase in phenols possibly occurred because Si structurally improves cell wall components, in which Ca is involved. Silicon forms complexes with structural cell polymers such as pectin and callose^60^, crosslinking with lignins and carbohydrates via associations with phenolic acids or aromatic rings^9^. This improves the structuring of the cell wall and decreases the extravasation of oxidative agents.

The antioxidant action of Si contributed to the increase in green colour index and leaf fluorescence quantum yield. Therefore, it reduced the damage caused to cell wall integrity, as can be seen from the lower electrolyte leakage index. Silicon addition increased Ca use efficiency and shoot dry matter production. Other studies have already demonstrated the positive effects of Si on the attenuation of Ca deficiency in rocket^21^, cabbage^20^, and *P. maximum* plants^28^, as well as in the hybrid Ipyporã (*B. ruziziensis* × *B. brizantha*)^28^. However, this is the first evidence that it also occurs in the forages *U. brizantha* cv. Marandu and *M. maximum* cv Massai.

### Implications and future perspectives

The practical implications obtained from the unprecedented information obtained in this research indicated that it is important to explore the relationship of Si in two agricultural scenarios that predominate in forage crops. The first, more frequent, refers to the fact that it is common to have stress due to nutritional deficiency of N, P, and Ca. In this condition, the benefits of Si in alleviating nutritional deficiency in the forages indicate the importance of this beneficial element for the sustainability of the cultivation of these species, as they are usually grown in low-fertility tropical soils. The second scenario, less frequent, refers to technified crops with frequent use of fertilisers without N, P, and Ca deficiency in the soil. Therefore, using Si could increase the nutritional efficiencies of these macronutrients, favouring or enhancing dry matter production.

It is now important to expand these studies to other forage species in other nutrients, to increase knowledge about the benefits of Si in forage plant physiology and nutrition.

## Conclusion

Silicon mitigates the effects of N, P, and Ca deficiency, improves antioxidant activity and photosynthesis rates, and N, P, and Ca use efficiencies and dry matter production of forage plants. The research emphasised the advantage of using Si to grow *U. brizantha* and *M. maximum* under N, P, and Ca deficiency, contributing to their sustainable cultivation.

## Supplementary Information

Supplementary Information.

Supplementary Table 1.
