# Supplementary material for: Silicon mitigates nutritional stress of nitrogen, phosphorus, and calcium deficiency in two forages plants
Source: Sci Rep. 2022 Apr 22;12:6611. doi: 10.1038/s41598-022-10615-z (PMC9033876; doi:10.1038/s41598-022-10615-z)
Supplement: Supplementary file 2 — Supplementary Table 1. [file 41598_2022_10615_MOESM2_ESM.docx]

**Table Supplementary 1.** Composition of the nutrient solution considered complete (CS) and the nutrient solution with reduced concentrations of nitrogen (-N), phosphorus (-P) and calcium (-Ca) in the absence (-Si) and in the presence of silicon (+Si) used in the experiment.

| Nutrient/  Element | CS-Si | CS+Si | -N-Si | -N+Si | -P-Si | -P+Si | -Ca-Si | -Ca+Si |
| --- | --- | --- | --- | --- | --- | --- | --- | --- |
|  | ---------------------------------- mmol L^-1^ ---------------------------------- | | | | | | | |
| Si | 0 | 2 | 0 | 2 | 0 | 2 | 0 | 2 |
| N | 7.5 | 7.5 | 3^a^  4.5^b^  6^c^ | 3^a^  4.5^b^  6^c^ | 7.5 | 7.5 | 7.5 | 7.5 |
| P | 0.5 | 0.5 | 0.5 | 0.5 | 0^d^  0.05^e^ | 0^d^  0.05^e^ | 0.5 | 0.5 |
| K | 3 | 3 | 3 | 3 | 3 | 3 | 3 | 3 |
| Ca | 2.5 | 2.5 | 2.5 | 2.5 | 2.5 | 2.5 | 0 | 0 |
| Mg | 1 | 1 | 1 | 1 | 1 | 1 | 1 | 1 |
| S | 1 | 1 | 1 | 1 | 1 | 1 | 1 | 1 |
|  | ---------------------------------- µmol L^-1^ ---------------------------------- | | | | | | | |
| Mn | 4.5 | 4.5 | 4.5 | 4.5 | 4.5 | 4.5 | 4.5 | 4.5 |
| B | 23 | 23 | 23 | 23 | 23 | 23 | 23 | 23 |
| Zn | 0.36 | 0.36 | 0.36 | 0.36 | 0.36 | 0.36 | 0.36 | 0.36 |
| Cu | 0.15 | 0.15 | 0.15 | 0.15 | 0.15 | 0.15 | 0.15 | 0.15 |
| Fe | 180 | 180 | 180 | 180 | 180 | 180 | 180 | 180 |
| Mo | 0.05 | 0.05 | 0.05 | 0.05 | 0.05 | 0.05 | 0.05 | 0.05 |

^a^Used from the 1^st^ to the 3^rd^ day after transplantation; ^b^Used from the 3^rd^ to the 6^th^ day after transplantation; ^c^Used from the 6^th^ to the 90^th^ day after transplantation; ^d^Used from the 1^st^ to the 30^rd^ day after transplantation; ^e^Used from the 31^th^ to the 90^th^ day after transplantation. Sources used to supply nutrients: N: KNO_3_, Ca(NO_3_)_2_.4H_2_O and NH_4_NO_3_; P: KH_2_PO_4_; K = KH_2_PO_4_, KNO_3_ and KCl; Ca: Ca(NO_3_)_2_.4H_2_O and CaCl_2_; Mg and S: MgSO_4_.7H_2_O; Mn: MnCl_2_.4H_2_O; B: H_3_BO_3_; Zn: ZnCl_2_; Cu: CuCl_2_; Fe: EDDHMA/Fe (6% Fe); Mo: H_2_MoO_4_.H_2_O.
